# Communicating treatment options to older patients with advanced kidney disease: a conversation analysis study

**DOI:** 10.1186/s12882-024-03855-w

**Published:** 2024-11-21

**Authors:** Lucy E. Selman, Chloe B. Shaw, Ryann Sowden, Fliss E. M. Murtagh, James A. Tulsky, Ruth Parry, Fergus J. Caskey, Rebecca K. Barnes

**Affiliations:** 1 Palliative and End of Life Care Research Group, Population Health Sciences, Bristol Medical School, University of Bristol, Canynge Hall, 39 Whatley Road, Bristol, BS8 2PS UK; 2 Wolfson Palliative Care Research Centre, Hull York Medical School, University of Hull, Hull, UK; 3 Dana-Farber Cancer Institute, Harvard Medical School Boston, Boston, MA USA; 4 Loughborough University, Loughborough, UK; 5 University of Bristol, Bristol Medical School, Bristol, UK; 6 North Bristol NHS Trust, Bristol, UK; 7 Nuffield Department Primary Care Health Sciences, University of Oxford, Oxford, UK

**Keywords:** Shared decision-making, Renal dialysis, Conservative treatment, Palliative care, Communication, Outpatient clinics, Hospital

## Abstract

**Background:**

Choosing to have dialysis or conservative kidney management is often challenging for older people with advanced kidney disease. While we know that clinical communication has a major impact on patients’ treatment decision-making, little is known about how this occurs in practice. The OSCAR study (Optimising Staff-Patient Communication in Advanced Renal disease) aimed to identify how clinicians present kidney failure treatment options in consultations with older patients and the implications of this for patient engagement.

**Methods:**

An observational, multi-method study design was adopted. Outpatient consultations at four UK renal units were video-recorded, and patients completed a post-consultation measure of shared decision-making (SDM-Q-9). Units were sampled according to variable rates of conservative management. Eligible patients were ≥ 65 years old with an eGFR of ≤ 20 mls/min/1.73m^2^ within the last 6 months. Video-recordings were screened to identify instances where clinicians presented both dialysis and conservative management. These instances were transcribed in fine-grained detail and recurrent practices identified using conversation-analytic methods, an empirical, observational approach to studying language and social interaction.

**Results:**

110 outpatient consultations were recorded (105 video, 5 audio only), involving 38 clinicians (doctors and nurses) and 94 patients: mean age 77 (65–97); 61 males/33 females; mean eGFR 15 (range 4–23). There were 21 instances where clinicians presented both dialysis and conservative management. Two main practices were identified: (1) Conservative management and dialysis both presented as the main treatment options; (2) Conservative management presented as a subordinate option to dialysis. The first practice was less commonly used (6 vs. 15 cases), but associated with more opportunities in the conversation for patients to ask questions and share their perspective, through which they tended to evaluate conservative management as an option that was potentially personally relevant. This practice was also associated with significantly higher post-consultation ratings of shared decision-making among patients (SDM-Q-9 median total score 24 vs. 37, *p* = 0.041).

**Conclusions:**

Presenting conservative management and dialysis as on an equal footing enables patient to take a more active role in decision-making. Findings should inform clinical communication skills training and education.

**Clinical trial number:**

No trial number as this is not a clinical trial.

**Supplementary Information:**

The online version contains supplementary material available at 10.1186/s12882-024-03855-w.

## Introduction

International guidance recommends that people with advanced kidney disease approaching kidney failure should not only be offered dialysis, but also Conservative Kidney Management (CKM) [1–3], which aims to delay disease progression and minimise adverse events, but without dialysis [4]. Although the evidence is not consistent, observational studies suggest that patients who opt for CKM live, on average, less long than those who opt for dialysis; however, any benefit is attenuated or lost for patients over 80 and for those with comorbidities [5, 6] and/or moderate frailty [7]. Survival benefit with dialysis must also be weighed against the likelihood of reduced quality of life, including functional and cognitive decline, and of higher symptom burden and risk of hospitalization and dying in hospital [6, 8]. 

The uncertain benefits and high burdens of dialysis for older people living with frailty and/or multimorbidity, mandate careful, person-centred decision-making support [9–11]. Patients may prioritise different outcomes to clinicians [12], focusing more on the impact a treatment has on their daily lives and goals [12–14]. How clinicians communicate information and support decision-making strongly influence treatment-choice [15–17]. Dialysis is often prioritised over CKM in education and information materials [18], and presented as the default option [9, 19, 20]. Treatment rates vary significantly; in 2012, the proportion of patients aged 75 + choosing CKM ranged from 5 to 95% across UK renal units [21]. This striking variability suggests that treatment decision-making is inconsistently guided by the evidence-base, and unlikely to be person-centred [22, 23], contrary to recommendations [1, 2]. 

To support shared decision-making in nephrology, clinician communication skills training is needed [9, 11, 16, 20]. Studies of real-life consultations provide evidence of what constitutes shared decision-making in practice, however such evidence is rarely used in existing training [24]. Recent studies of treatment decision-making in advanced kidney disease in the Netherlands [25] and Australia [18, 26] have collected recordings of real-life consultations. While these studies provide novel insight into what actually happens in these encounters, the analytic approach in these studies was insufficiently fine-grained to allow them to elucidate how clinicians frame treatment options or the implications of different communication practices for patient involvement in treatment decision-making.

Conversation Analysis (CA) is a distinctive, highly empirical method for studying medical interactions, used to identify in fine-grained detail problems in communication as well as their solutions [27], including in the context of discussing treatment options. The method examines not only what is said (i.e. language), but how it is said [28–30]. CA studies have linked communication practices to patient-relevant outcomes that are both internal and external to the conversation, providing important evidence to inform person-centred care and training that changes clinician behaviour [31, 32]. Using CA, this study aimed to systematically identify renal clinicians’ different approaches to presenting CKM, describe the key interactional features of these approaches, and examine their implications for patient involvement in treatment decision-making.

## Methods

### Study design

The OSCAR study (Optimising Staff-Patient Communication in Advanced Renal disease) was a mixed methods study to understand how renal clinicians communicate with patients with advanced kidney disease regarding their treatment options. Renal outpatient consultations were video-recorded and analysed using the method of Conversation Analysis; a fine-grained observational approach to studying verbal and nonverbal components of communication. Questionnaire data were collected from patients, companions (where present) and clinicians, post recording. The questionnaire included the 9-item Shared Decision-Making Questionnaire (SDM-Q-9) [33] (see Appendix 1), a validated measure of shared decision-making in clinical interactions, using a 6-point scale (from 0 = completely disagree to 5 = completely agree). Clinicians were also invited to complete a post-consultation questionnaire which included the 9-item SDM-Q-Doc, the clinician version of the SDM-Q-9 [34].

Data were collected from July 2021 to January 2023. A project Patient and Public Involvement group contributed throughout the study. Reporting follows the Standards for Reporting Qualitative Research [35], adapted for a CA study [36].

### Participants and setting

Recruitment was via four renal units in the UK, purposively selected to represent a range of service models and CKM treatment rates (Table 1). Patients were eligible if they were age *≥* 65 and had an eGFR of ≤ 20 mL/min within the last 6 months, and had chronic kidney disease. After assessment of eligibility, information was sent to them in the post in advance of the clinic, enabling patients to opt in or out and have their questions answered prior to their clinic appointment. A two-stage consent process was used, with consent sought in-person by a member of the research team prior to entry to the clinic room, and re-confirmed after the consultation. Representing the range of service models in the four renal units, patients may have been attending outpatient clinics, satellite outpatient clinics, ‘low clearance’ clinics (specialised clinics for people approaching kidney failure), or joint-run renal and palliative care clinics. People who accompanied patients during their consultations (‘companions’) were also included. Included clinicians were doctors or nurses who met patients with CKD with an eGFR of ≤ 20 and discussed treatment options with them. At each site, we aimed to recruit approximately 6 clinicians with diverse characteristics, and to record 4–6 patient consultations per clinician, totalling 40–90 h of recorded data. We estimated that this would result in > 80 survey responses, sufficient for exploratory analyses. For sampling, screening and recruitment details, see Supplementary Material 1.

**Table 1:** Characteristics of sites and populations served

	Site 1	Site 2	Site 3	Site 4
Presence of a low clearance clinic	Not at main hospital, but at some satellite sites	Yes	Yes	Yes
Black, Asian and minority ethnic patient ethnicity (%) (UK Renal Registry. 2022) [51]	12.1 South Asian 3.0% Black 6.7% Other 2.4%	10.2 South Asian 6.2% Black 0.6% Other 3.4%	3 South Asian 3.0% Black 0.0% Other 0.0%	57.7 South Asian 21.0% Black 26.2% Other 10.5%
Percentage of patients 75 + receiving CKM (calculated from CKMAPPS data, 2012)[21]	16%	28%	1–9%	45%
Number of patients with CKD 5 aged 75 + in 2016^1^	350	82e	120e	318
Number (%) of CKD 5 patients aged 75 + who were on CKM in 2016^1^	182 (52%)	36e (44%)	15-20e (13–17%)	115 (36%)

^1^Data collected directly from sites via e-mail

### Data collection

To record the consultations, two GoPro Hero 9 cameras and/or Dictaphones were set up in the consultation room, in unobtrusive places. Recording devices were set to record by RS/LS prior to the consultation start. RS is a healthcare researcher and speech and language therapist; LS is an experienced social scientist with expertise in serious illness research. Researchers were not present during recording except in two instances where requested by the patient. Post-consultation, patients completed a questionnaire either at home, online, or by telephone with RS. Patients and companions who completed questionnaires received a £10 voucher.

### Data analysis

#### Recorded consultations

Consultations were analysed using applied Conversation Analysis (CA), a well-established method for analysing clinical interactions, guided by communication challenges faced by clinicians [37]. Addressing these types of problems typically involves the collection of both observational, interactional and quantitative outcomes data. CS screened all recorded consultations to identify segments where clinicians presented both treatment options, enabling systematic comparison of how options were framed. The identified segments were pseudonymised and transcribed in fine-grained detail using the Jefferson Transcription System [38] and analysed for their position (e.g., where in the consultation they arose); the observable actions being implemented, and their lexical design; and the subsequent turn-by-turn trajectory, including how the patient responded [39]. Initial findings were discussed and analysed in regular group data sessions (CS, RS, LS, RB, RP), with multi-disciplinary co-authors and PPI members. Previously established conversation analytic findings were used as ‘tools’ in the analysis.

Communication practices found recurrently across the data were then tested for association with the post-consultation questionnaire scores.

Given that a patient’s ultimate decision is (a) often made over multiple time points and (b) can change over time, the analysis focuses specifically on the decision-making process, and linking empirical findings of conversational practices to patient reflections of that process, rather than what decision was ultimately made.

### Questionnaires

SDM-Q-9/Doc total scores were transformed to a score of 0-100 in line with recommended practice to make the scores more intuitive to interpret [33]; higher scores represent a higher rating of shared decision-making [33]. Median scores for questions were compared between groups according to how treatment options were presented, using a non-parametric Median Test.

### Ethics statement

Ethical approval was granted by the HRA London-Bromley Research Ethics Committee (21/LO/0280). Participants gave written informed consent.

## Results

### Dataset characteristics

110 outpatient consultations were recorded: 105 video-recorded (in-person consultations), 5 audio-recorded (4 telephone consultations, 1 in-person). Consultations involved 94 patients, 40 companions and 38 clinicians (Table 2). Consultations were with nephrology consultants (*n* = 86/110); nephrology registrars or junior doctors (*n* = 6), renal education nurses (*n* = 13), and both palliative care nurses and renal education nurses (*n* = 5). Patients completed surveys for 85/110 consultations.

**Table 2:** Patient and clinician demographics – overall and by clinician option-listing strategy

**Patient Demographics**	**Participant grouping**	**Age in years (mean, (SD); range)**	**Sex (n)**	**Ethnicity (n)**	**eGFR in (ml/ min/1.73m**^2^) **(mean, (SD); range)**	**Frailty score**^1^ **(mean, (SD); range)**	**Comorbidity score**^2^ **(mean (SD); range)**
	Total (*n* = 94)	77 (7.32); 65–97	Female: 33 Male: 61	White: 73 Asian: 8 Black: 5 Mixed: 1 Other^7^: 7	15 (3.69); 4-23^3^	1.6 (1.03); 0–4	1 (0.65); 0–2
	CKM presented as a main treatment option (*n* = 6)	80 (3.19); 74-83.25	Male = 5 Female = 1	White = 5 Other^7^ = 1	17 (2.48); 15–20	1.67 (1.21); 0–3	0.67 (0.52); 0–1
	CKM presented as a subordinate option (*n* = 15)	74 (4.09); 68.67–82.92	Male = 14 Female = 1	White = 13 Other^7^ = 2	18 (2.43); 15–23	1.13 (0.74); 0–3	1 (0.53); 0–2
**Clinician Demographics**	**Participant grouping**	**Age in years (mean, (SD); range)**	**Sex**	**Ethnicity**	**Role**		
	Total (*n* = 38)	47 (8.73); 31.37-65.39^4^	Female: 21 Male: 17	White: 21 Asian: 8 Black: 2 Chinese: 1 Other^7^: 2 Did not indicate: 4	Consultant: 23 Registrar or junior doctor: 4 Nurse: 11		
	CKM presented as a main treatment option (*n* = 5)^5^	44 (3.72); 41.34–50.34	Female: 5 Male: 1	White: 4 Asian: 1	Consultant: 4 Nurse: 1		
	CKM presented as a subordinate option (*n* = 9)^6^	49 (7.35); (39.10 − 58.42)	Female: 5 Male: 4	White: 5 Asian: 3 Did not indicate: 1	Consultant: 7 Nurse: 2		

^1^WHO Performance Score [52]

^2^Davies Grade [53]

^3^All patients had an eGfR of ≤ 20 in the six months prior to recording but may have had a higher reading around the time of recording

^4^3 missing

^5^5 clinicians recorded (1 recorded twice)

^6^9 clinicians recorded (4 recorded more than once)

^7^“Other” ethnicity includes Arab, and any ethnic group other than: Asian or Asian British, Black, Black British, Caribbean or African, Mixed or multiple ethnic groups, or White, as categorised by the 2021 UK Census

We identified 23/110 conversations in which kidney failure treatments were presented. In 3/23 conversations, there was no mention at all of CKM or ‘not having dialysis’. In 20 consultations, options were listed that included CKM, forming the core collection for analysis (6 h, 41 min of data). These conversations included 16 patients (three patients were recorded more than once); companions were present in 9/20. 17/20 conversations involved consultant doctors; 4/20 (specialist) nurses. There were a total of 21 segments of interaction where options were presented (in one consultation, CKM was presented at two different times, in two different ways).

### Participant characteristics

In total, 94 patients and 38 clinicians participated. Patients had a mean age of 77 (range 65–97), included 61 males and 33 females, with a mean eGFR of 15 mL/min (range 4–23), mean frailty score of 1.6 (0–4) and mean comorbidity score of 1 (0–2). Clinician participants had a mean age of 47 (range 31–65); 21 were female, 17 male. Further demographic data are presented in Table 2.

### Findings

#### Approaches to presenting options

We identified two approaches to framing CKM when presenting treatment options. The less common approach (6/21) presented CKM as a main option alongside dialysis. The more common approach (15/21) presented CKM as a subordinate option. Patient and clinician characteristics are compared by approach in Table 2; there were no clear differences between groups. The length of consultation did not differ according to approach used (mean 23 min). Features of the two approaches to framing CKM are explicated and exemplified next (summarised in Table 3), before outlining the interactional consequences of these approaches for patient engagement.

**Table 3:** Recurrent elements of the two approaches to presenting CKM

CKM as a main option	CKM as a subordinate option
CKM is framed as a clear treatment option • Introduced as part of the main decision-making sequence • Labelled as a clear treatment option and CKM • Details provided of what is involved • CKM is not framed as only relevant or preferable to a minority of patients • The potential benefit(s) of CKM/limitations of dialysis are described	CKM is not framed as a clear treatment option • Appended to the main decision-making sequence • Not labelled as a clear treatment option/CKM but as an omission (not having dialysis) • Minimal/no details provided of what is involved • Not having dialysis maybe ruled out as ‘not for you’ • CKM is framed as relevant or preferable only to a minority of patients • CKM is not clearly presented as having benefit to the patient

##### CKM as a main option

**Fig. 1:**
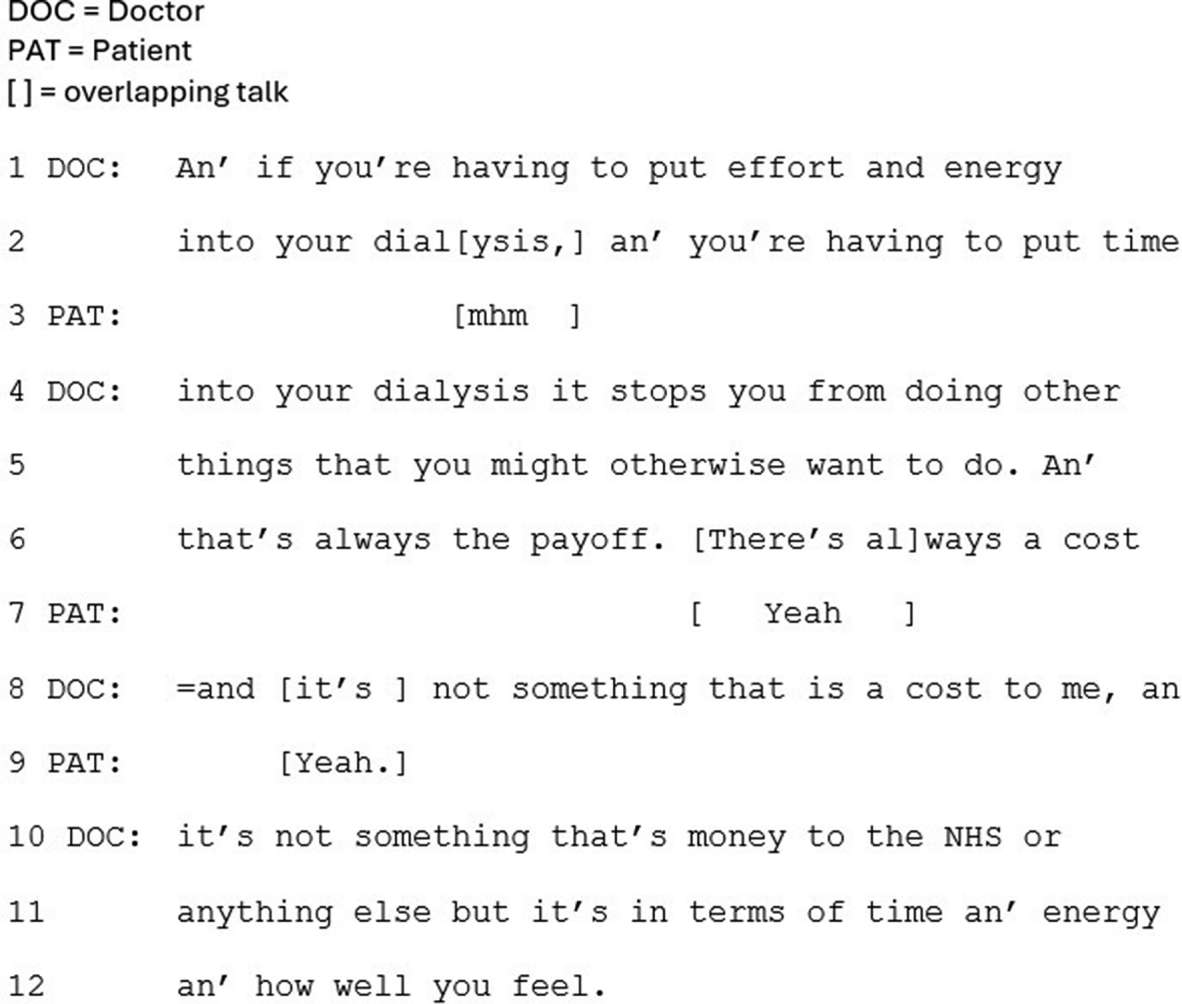
Extract 1

Less commonly, CKM was presented in a way that frames it as a main option (*n* = 6). Firstly and crucially, in this approach CKM is framed as being of potential benefit to the patient in terms of quality of life, in all cases. One of the ways this is achieved is through presenting the disadvantages of dialysis; its time burden, and how it makes people feel. In Fig. 1, the doctor refers to the ‘cost’ (line 8) associated with dialysis, which “stops you from doing other things that you might otherwise want to do” (lines 4–5).

**Fig. 2:**
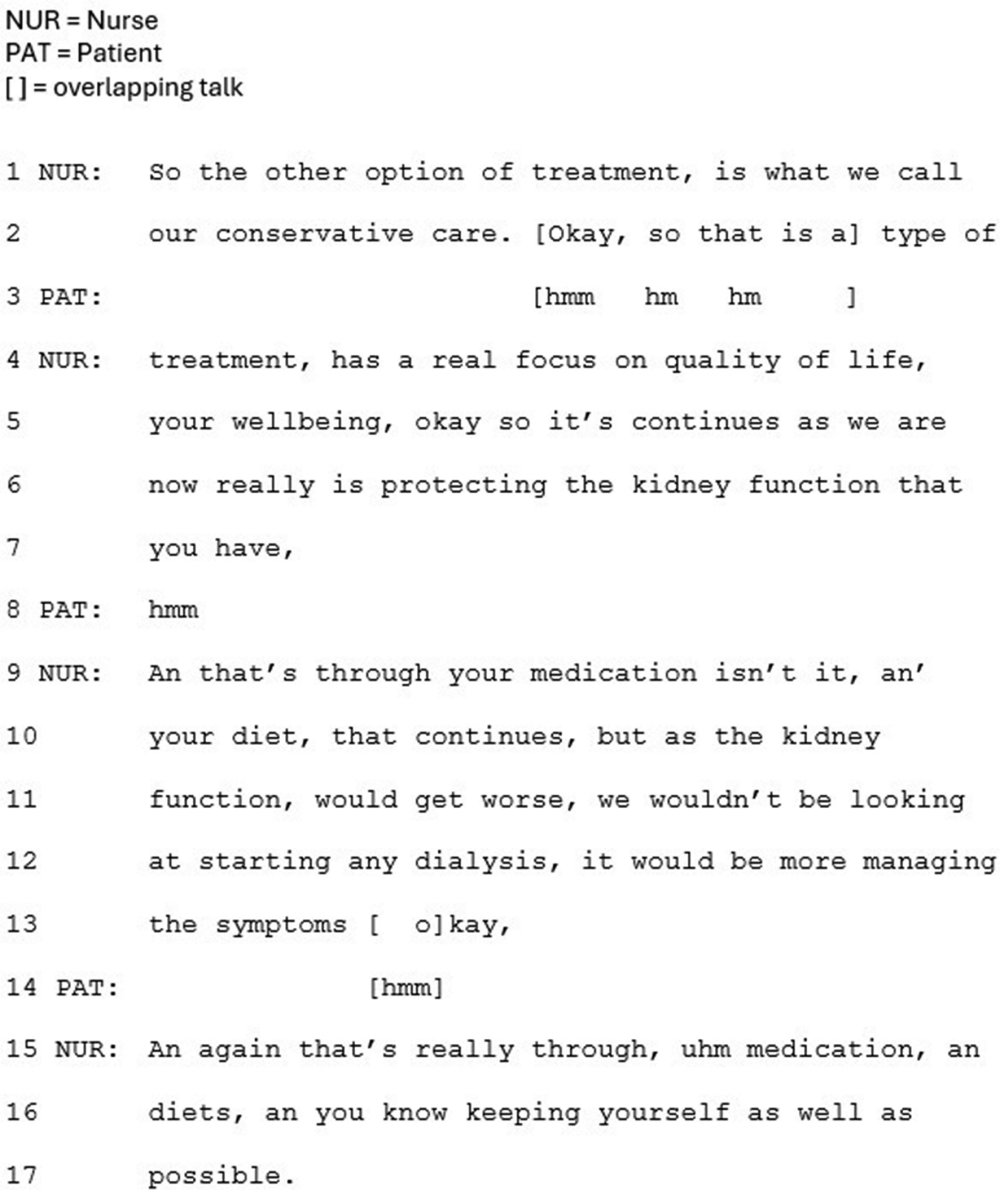
Extract 2

**Fig. 3:**
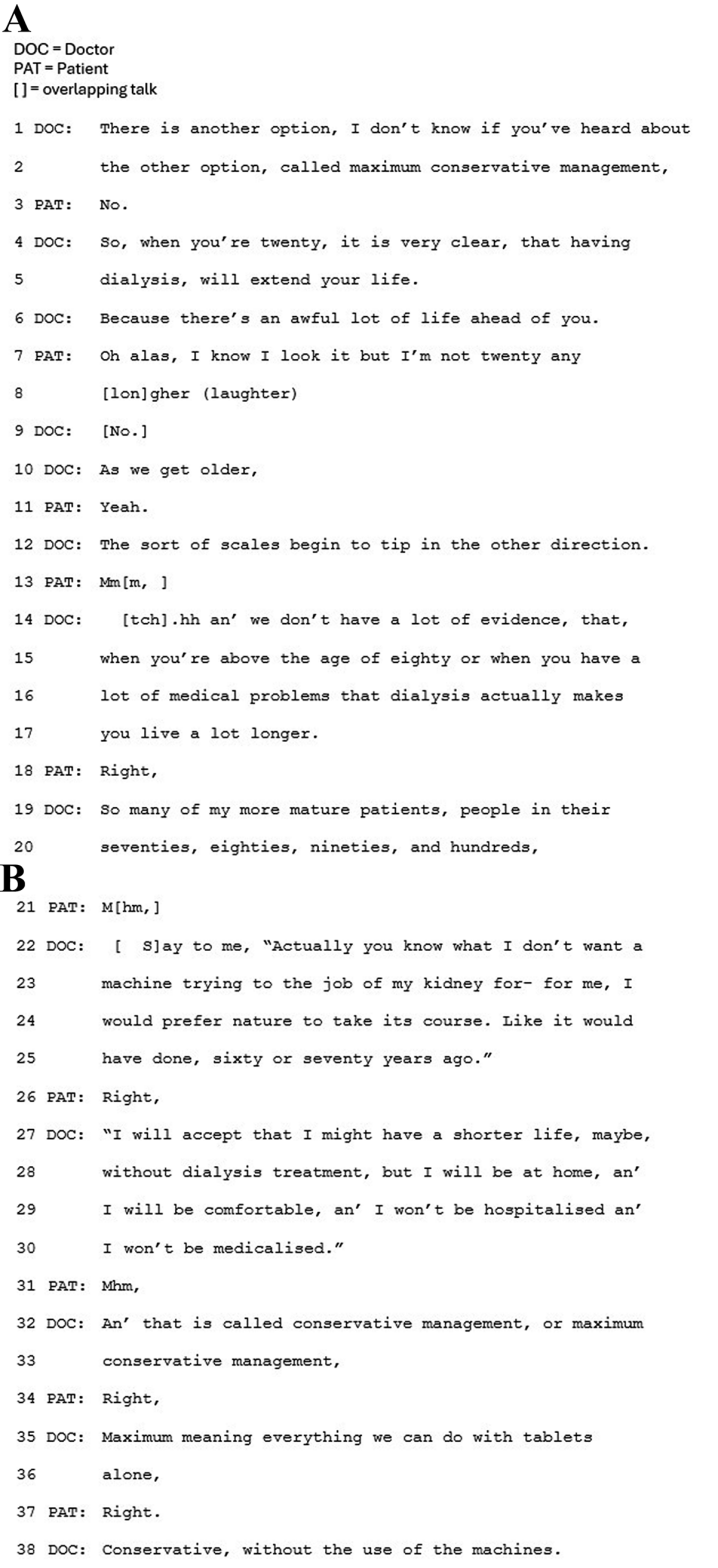
Extract 3

The benefit of CKM is also framed positively in terms of the impact upon the patient’s quality of life (e.g. Figure 2, lines 4–5). In all cases, the clinician refers to dialysis’s potentially limited benefit to length of life, for older people in particular, and research evidence is referred to either explicitly as ‘evidence’ (Fig. 3, line 14), or implicitly by referring to ‘what is known’.

Secondly, within this approach CKM tends to be introduced as part of the main decision-making sequence. This is shown in Figs. 2 and 3 (line 1 for both), where CKM is introduced as ‘the other/another option.’ As such, it features as one in a list of options – underscored by the explicit labelling of it as an ‘option.’ Furthermore, with this approach clinicians recurrently do not frame CKM as relevant or preferable to only a minority of patients. In Fig. 3, the clinician even presents CKM as a popular choice (line 19). These features together frame CKM as one of the main options to choose from.

In this approach CKM is also more clearly framed as an active treatment. It is more frequently labelled as CKM, and as a treatment, with details provided of what is involved. For example, the doctor in Fig. 3 refers to “maximum, meaning everything we can do with tablets alone” (lines 35–36), and in Fig. 2, the nurse explicitly labels ‘conservative care’ as treatment (lines 2 and 4), going on to specify that ‘treatment’ involves medication and diet.

##### CKM as a subordinate option

**Fig. 4:**
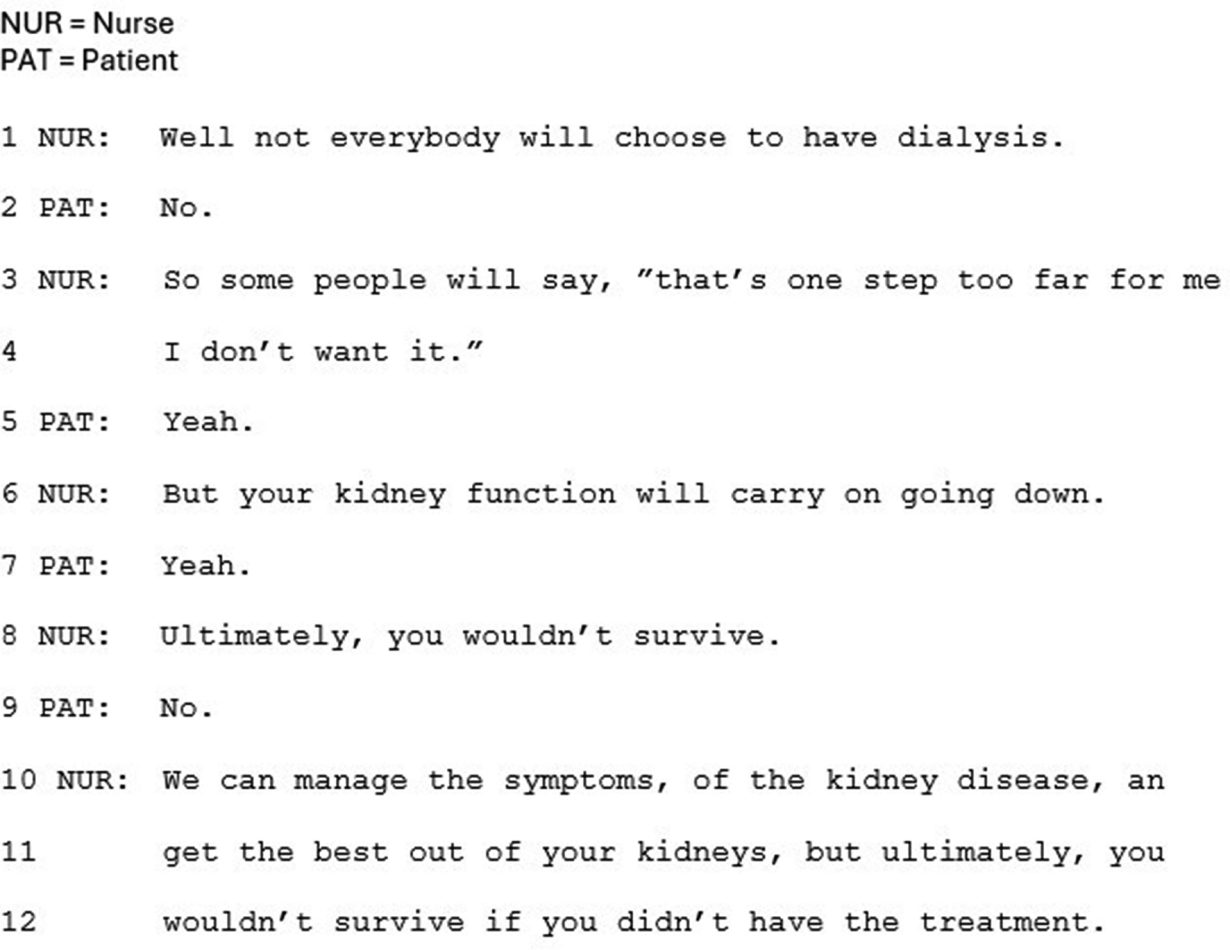
Extract 4

In the alternative approach, firstly, and most crucially, CKM is not presented as having clear potential benefit for the patient: the disadvantages of dialysis are not explicated, and neither are CKM’s potential advantages. For example, in Fig. 4 (lines 3–4), the only rationale for choosing not to have dialysis is that dialysis is “one step too far.” Significantly adding to the weighting towards dialysis, not having it is equated with not surviving (lines 11–12).

**Fig. 5:**
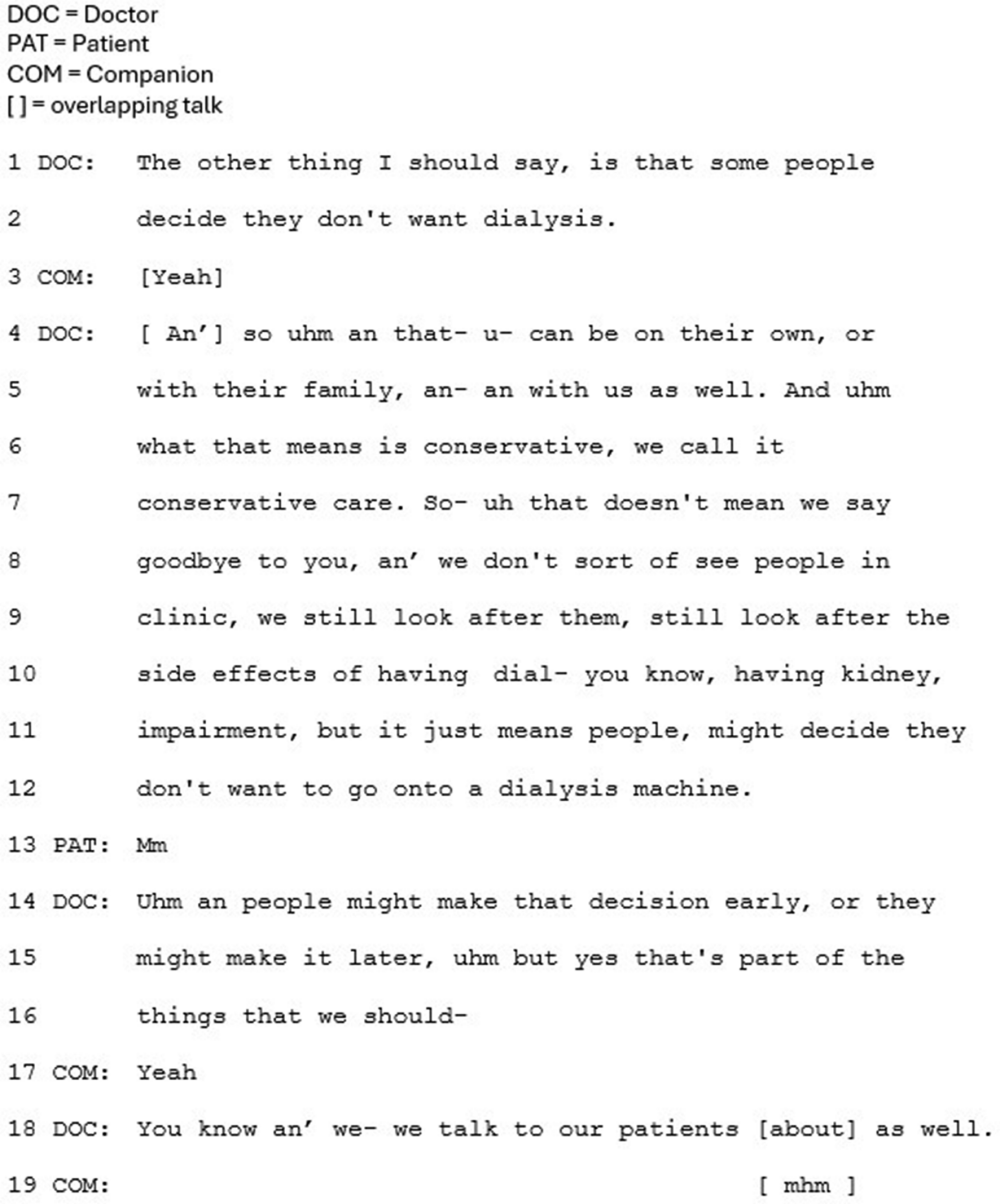
Extract 5

In Fig. 5, when the option of CKM is presented, no rationale for choosing not to have dialysis is provided. Earlier in that conversation, the clinician introduced some disadvantages of dialysis, referring to it as being “quite a big physical burden in terms of how people feel.” However, this disadvantage is not presented as a reason for not choosing dialysis and/or for choosing CKM instead but is raised just before introducing dialysis access.

**Fig. 6:**
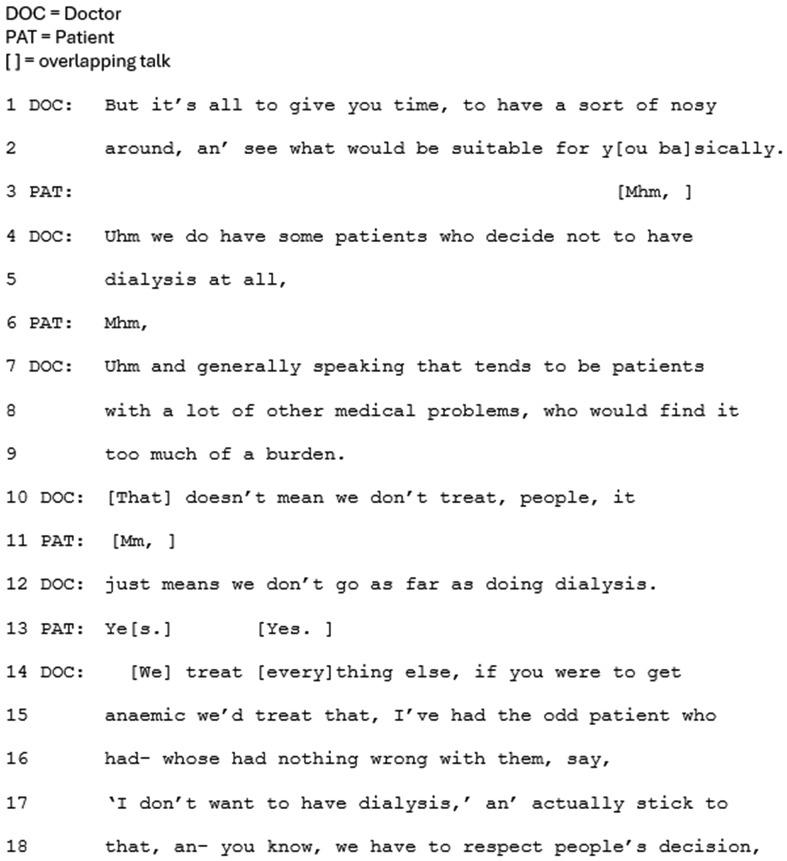
Extract 6

In this approach CKM is recurrently presented as the last option, after dialysis treatments have been presented. Whilst being presented last may not in and of itself subordinate an option, the positioning of CKM as something extra, beyond the main options, does. The option of CKM is regularly appended to the main list of treatment options in this approach (e.g. Figure 5). Prior to the extract in Fig. 5, the clinician has been closing the conversation by deferring the need for a decision: *“go home, think about it, probably come back again you know, an’ then kind of hopefully come to a decision, a bit later down the line.”* The option of CKM is then introduced, framed as something extra, rather than explicitly labelled as another option: “The other thing I should say” (line 1). The ‘should’ suggests the clinician is morally obliged to raise this option, indicating a reluctance to raising it, and implying a less favorable option. Not overtly labelling CKM as an option is recurrent in this approach (e.g. Figure 4) which, together with the appended positioning, subordinates CKM as a treatment option. This notion is underscored by framing CKM as relevant or preferable to only a minority of patients: ‘some people’ (Fig. 4, line 3; Fig. 5, line 1; Fig. 6, lines 4–55). Whilst this validates CKM as an option, it frames this choice as less common than choosing dialysis, and no longer ‘mainstream.’ The reference to ‘some people’ also leaves it to the patient to make the inference that the option is relevant to themselves personally.

Compared to dialysis, CKM is also much less frequently labelled explicitly as CKM or even as a treatment. It is more often referred to by negation; as ‘not having dialysis’. Indeed, in Fig. 4 (line 12), dialysis is equated with “the treatment”, implying that not having dialysis is not having treatment. Furthermore, in these cases, ‘not having dialysis’/CKM is not referred to as an option – a notion underscored by the often minimal information provided about what CKM entails. Clinicians often deal explicitly with the possible negative assumption that the patient won’t be cared for with this approach, as in Fig. 5 (lines 7–8: “that doesn’t mean we say goodbye to you”), to suggest something positive; that the patient will still be cared for (see also Fig. 6, line 10). This form of understatement, or litotes [40], is often used to indirectly refer to something delicate. Here it has the effect of avoiding presenting CKM as inherently positive.

In a sub-collection of cases (*n* = 7), the disadvantages of dialysis and rationale for choosing CKM are presented but they are ruled out as only being relevant to people who differ from the patient, e.g. older patients and/or those with more health problems (Fig. 6, lines 7–9). So whilst potential benefits of CKM are presented, they are then framed as not relevant to this patient. Notably, in only two cases for this approach do clinicians refer to research evidence about how dialysis impacts length of life; in both cases, this is framed as not relevant to the patient.

#### The interactional consequences of the alternative approaches

**Fig. 7:**
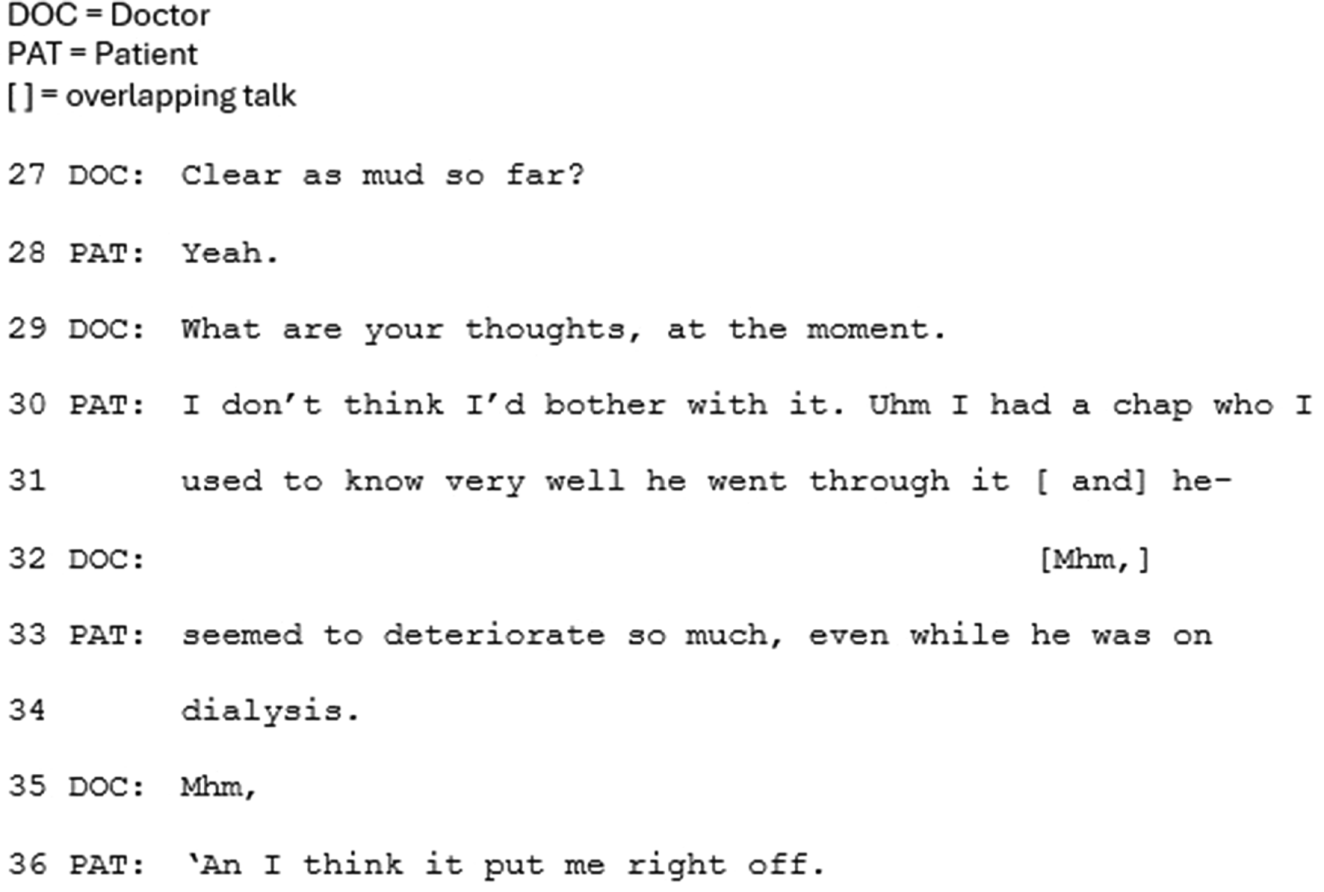
Extract 7 (Extract 1 continued)

When CKM is presented as a main treatment option, more opportunities tend to be provided for the patient to ask questions about CKM or not having dialysis and to provide their perspective about CKM, and therefore to consider it as a relevant option. The patient’s perspective is frequently invited through explicit questions (sometimes termed ‘Patient View Elicitors’ [29]) and less explicitly through not moving the conversation on but providing an interactional slot for the patient to ask questions or provide their perspective about CKM. Following this approach, the patients tended to evaluate not having dialysis as a relevant option in the conversation. This is demonstrated in Fig. 7, below. After discussing the disadvantages of dialysis, the clinician explicitly invites the patient’s perspective (Line 29). The patient then asserts his preference against having dialysis.

**Fig. 8:**
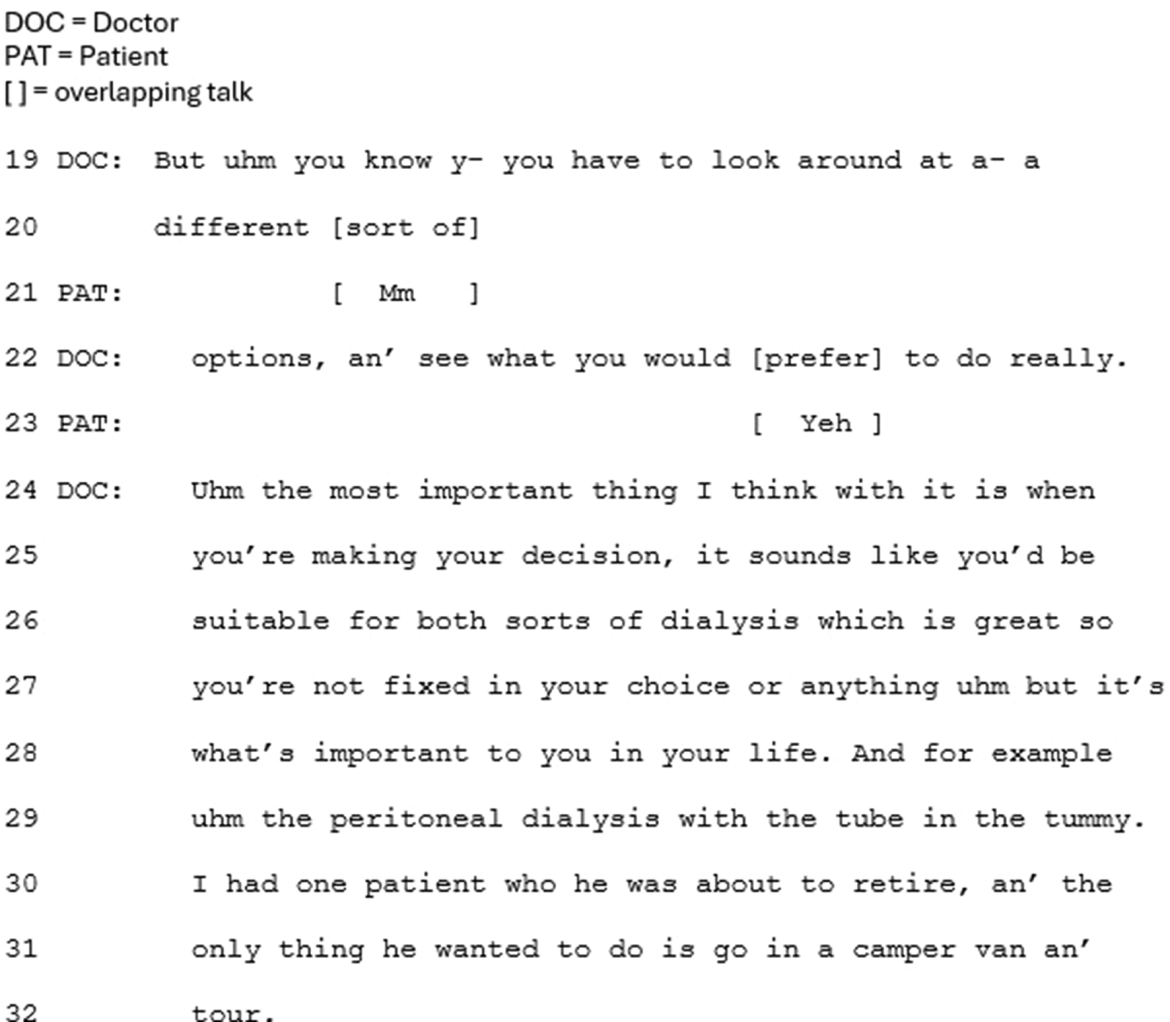
Extract 8 (Extract 6 continued)

In contrast, when CKM is presented as a subordinate option, the clinician tends to move the sequence on, away from the ‘option’ of CKM, without first having asked the patient about their perspective or inviting their questions regarding CKM. Furthermore, in these cases patients do not positively evaluate CKM or orient to it as a viable option. In Fig. 8, which follows directly on from Fig. 6, the clinician moves the conversation away from the option of CKM. She does not provide a slot for the patient to assess this option, but instead brings the focus of the conversation back to the patient’s suitability for the two dialysis options, which she positively assesses as ‘great’ (line 26) (see Table 4 for interactional implications by approach).

**Table 4:** The interactional implications of alternative approaches to presenting CKM/not having dialysis within the consultation

		*1. Clinician’s subsequent action towards CKM/ not having dialysis*			*2. Patient’s subsequent action towards CKM/ not having dialysis*		
		**Clinician invites patient’s perspective and/or invites patient to ask questions**	**Clinician moves on to other matters without patient having expressed their perspective / asked question(s)**	**Patient speaks next without having been explicitly invited to ask questions or give their perspective [categorised in column 2]**	**Patient positively evaluates CKM/ not having dialysis as a relevant option for them**	**Minimal uptake/no positive evaluation of CKM as an option**	**Patient continues to give reasons for not wanting dialysis**
*Framing of CKM/ not having dialysis*	**CKM presented as a main treatment option (n = 6)**	4/6	0/6	2/6	4/6	1/6	1/6
	**CKM presented as a subordinate option (n = 15)**	2/15 (42, 1201 – sessions set up for gaining info about decision)	8/15	5/15	0/15	14/15	1/5 (in a way that shows push towards dialysis)

#### Association with post-consultation shared decision-making scores

When clinicians presented CKM as a main (*n* = 6) rather than subordinate (*n* = 9) option, median total SDM-Q-9 scores were significantly higher (*p* = 0.041), as were the medians for questions 3–7 (Q3: *p* = 0.04; Q4: *p* = 0.011;Q5: *p* = 0.002; Q6: *p* = 0.041; Q7: *p* = 0.041) (Table 5).

**Table 5:** Questionnaire results for patients and clinicians according to option-listing approach

	Questionnaire	CKM a subordinate option (*n* = 9) [Median and Range]	CKM a valid option (*n* = 6)^1^ [Median and Range]	Median Test for K samples [*p* values]
Patient responses	**SDM-Q-9 Total score** **(9 items)**	24 (0–36) Transformed data: 53.33 (0–80)	37 (6–45) Transformed data: 82.23 (13.33–100)	0.041
	My clinician (MC) made clear that a decision needs to be made.	3 (0–4)	2 (0–5)	0.329
	MC wanted to know exactly how I want to be involved in making the decision.	3 (0–5)	4.5 (3–5)	0.235
	MC told me that there are different options for treating my medical condition.	3 (0–5)	5 (0–5)	0.041
	MC precisely explained the advantages and disadvantages of the treatment options.	3 (0–5)	5 (0–5)	0.011
	MC helped me understand all the information.	3 (0–4)	5 (0–5)	0.002
	MC asked me which treatment option I prefer.	2 (0–5)	4.5 (0–5)	0.041
	MC and I thoroughly weighed the different treatment options.	2 (0–4)	5 (0–5)	0.041
	MC and I selected a treatment option together.	2 (0–4)	3 (0–5)	0.329
	MC and I reached an agreement on how to proceed.	2 (0–4)	4 (1–5)	0.315
Clinician responses	**SDM-Q-Doc Total score**	31 (24–40) Transformed data: 68.89 (53.33–88.89)	29.5 (19.1–34) Transformed data: 64.56 (42.44–75.56)	1.00
	I made clear to my patient that a decision needs to be made.	3 (0–5)	3 (0–4)	0.603
	I wanted to know exactly from my patient how he/she wants to be involved in making the decision.	3 (3–5)	3.5 (3–4)	0.584
	I told my patient that there are different options for treating his/her medical condition.	5 (2–5)	4 (2.1-4)	0.082
	I precisely explained the advantages and disadvantages of the treatment options to my patient.	3 (2–5)	3 (1–3)	0.237
	I helped my patient understand all the information.	4 (3–5)	3 (1–3)	0.103
	I asked my patient which treatment option he/she prefers.	4 (2–5)	4.5 (3–5)	0.538
	My patient and I thoroughly weighed the different treatment options.	3 (2–5)	3 (1–4)	1.00
	My patients and I selected a treatment option together.	2 (0–5)	2.5 (1–4)	0.584
	My patient and I reached an agreement on how to proceed.	4 (3–5)	4 (3–5)	1.00

^1^For one patient, one survey was completed following two separate consultations on the same day, where CKM as a main option approach was used in both. The results were linked to each consultation separately

There were no significant differences between post-consultation SDM-Q-Doc scores according to option-listing approach.

## Discussion

This study provides the first fine-grained analysis of the relationship between the conversational practices used by renal clinicians and patients’ engagement with treatment options and ratings of shared decision-making. As such, our findings illuminate how clinicians’ communication about treatment options influences patient involvement and treatment decisions.

We found that dialysis treatments tended to be presented as the default treatments for advanced kidney disease, with CKM presented as a subordinate option. A Dutch study also reports the same imbalance in how the benefits and harms of kidney failure treatments are communicated [25]. Our research goes further by considering the interactional consequences of this imbalance. By listing CKM as one of several options; labelling it as a treatment option; and framing it as active treatment with potential benefit to this particular individual, patients were more likely to engage with the option of CKM or not having dialysis during the conversation. That is: the clinician was more likely to invite the patient’s perspective and questions, and the patient was more likely to evaluate CKM out loud as a *relevant* option for them, whether or not this was their ultimate choice. In contrast, when CKM was presented as a subordinate option, the clinician tended to move the sequence on, away from the option of CKM, and there was minimal uptake from the patient and no positive evaluation of CKM as an option.

The CKM as a valid option approach was associated with significantly higher patient-reported shared decision-making scores compared with the ‘CKM as subordinate option’ approach. The latter was associated with an average score of 53.3/100, comparable to scores in a US study of adults aged 70 + years with advanced kidney disease, which found a mean score on the SDM-Q-9 of 52/100, described as suboptimal (with middle values equating to ‘somewhat disagree’ and ‘somewhat agree’) [23]. The specific items that we found to be significantly higher for the approach presenting CKM as a main option ask whether the clinician: told the patient about different treatment options (Q3), explained the benefits and limitations of options (Q4 and 7), and specifically asked about their treatment preference (Q6) (see Appendix 1 and Table 5). Through the empirical analysis of actual conversations we found that all of these conversational activities are indeed absent or limited for Approach 2, in which CKM is framed as a subordinate option. This supports evidence from haemodialysis patients that being informed about treatment options in a balanced way results in higher shared decision-making scores [17]. Our finding of no significant difference in scores for the clinician self-reports of shared decision-making suggests that clinicians may not be aware of the influence their communication approach has on patient-reported experiences of shared decision-making.

Presenting CKM as an option takes one step towards shared decision-making – improving on the three cases we identified in which CKM was not mentioned as a treatment option at all, echoing previous research [25, 26]. But providing options is not enough to engage patients in shared decision-making; clinicians need to do more [29]. Whilst clinicians provide options in both approaches we identified, it is *how* these options are presented which impacts patients’ engagement with them in the conversation. Our findings thus might help to explain the gap between what clinicians often say (that CKM is available), and what patients often report (that they haven’t heard of CKM) [41]. 

Our findings highlight the importance of clinicians clearly detailing the advantages and disadvantages of both treatment pathways, supporting evidence that a patient only assesses a future course of action as an option when it is framed as having potential benefit to them [29]. When CKM was framed as a main option, the benefits of CKM and limitations of dialysis were described; this was not the case when it was framed as a subordinate option, and there was also no reference to existing best outcome evidence for each option. This might indicate under-developed communication skills, gaps in clinician knowledge and/or difficulties in translating evidence into practice. By not presenting or downplaying the disadvantages of dialysis, clinicians may be avoiding talk about prognosis and the potential implications of choosing dialysis for patients’ end-of-life care [19, 42]. Fully discussing these implications with patients could help reduce unwanted, futile care [43] and decisional regret [44]. Clinicians may also be concerned about starting up longer conversations, when framing CKM more equitably, and yet we found no significant difference in the length of consultation according to the approach used.

Findings also highlight the importance of using the term ‘Conservative Kidney Management’ (or similar) as an affirmative label [19, 28], rather than simply ‘not having dialysis’, which presents a negative choice or absence of something. Findings from a recent Discrete Choice Experiment likewise demonstrated that older patients prefer a ‘treatment’ option rather than ‘no treatment’ option [45]. Patients are receptive to CKM, when it is framed as an active rather than passive treatment [13]. Ensuring clinicians present CKM as active treatment which can help achieve specific patient goals and/or align with their values, is therefore crucial [19, 41]. 

These findings begin to specify what person-centred support with treatment decision-making looks like in practice; for example, inviting patients to assert their perspective or ask questions, and framing options as having potential benefit to the patient, so that they may then consider an option as relevant to them (whether they ultimately choose it or not). When clinicians present CKM as a subordinate option, they may be guiding patients towards the treatment they think they will choose, based on their experience and knowledge of the patient [25]. However, this may prevent patients from fully exploring the range of options available to them in time to choose treatment that best fits their preferences. For example, starting dialysis transforms the non-dialysis treatment option from CKM into dialysis discontinuation. Since initiation of dialysis may be associated with loss of residual kidney function [46–48], even early discontinuation may be associated with worse survival outcomes compared with CKM, had that been chosen in the first place. A method of stratifying the conversational approach according to specific clinical variables would likewise limit the options available to patients, particularly as these decisions usually occur in advance of when a choice is enacted, during which time these variables are likely to change. Arguably, kidney clinicians should be explicitly asking patients what matters to them [10]. Our analysis of how CKM can be framed as a main and valid option alongside dialysis contributes to a growing body of direct evidence of the impact of clinicians’ presentation of treatment options on shared decision-making [30], informing theoretical concepts of patient-centred care and decision-making support [24]. 

Whilst we have identified two alternative approaches to presenting CKM, with alternative trajectories for patient engagement, these approaches comprise a number of components or practices which may be used to varying degrees along a cline. For example, in Fig. 5, a CKM as subordinate option case, CKM is labelled as ‘Conservative Care’ rather than simply ‘not having dialysis.’ Conversely, clinicians presenting CKM as a main option may still display some orientation to dialysis as default, reflecting and contributing to widespread assumptions that advanced kidney disease leads to dialysis. The crucial component which distinguishes the two approaches is whether CKM is presented as having potential benefit to the patient.

Study strengths include recording a large sample of consultations with a diverse group of clinicians, and following a rigorous screening process to identify specific conversations in which treatments were listed (see Supplementary Material 2). The analysis is based on a limited number of cases but provides evidence of a robust pattern identified across a variety of settings and practitioners. Whilst our survey results reveal an indicative and novel relationship between communication practices and patient-reported outcomes, results should be interpreted with caution due to the number of consultations included in this analysis. Consultation extracts were regularly analysed in group data sessions, but audiovisual data were not independently categorized by another researcher. Finally, a risk of video-recording consultations is that parties might not communicate in the usual way. However, most people feel only slightly or not at all influenced by video-recording [49] and patients do not feel that it alters the treatment they receive [50]. 

## Conclusions

In conclusion, in our study, when talking to older people, kidney doctors and nurses more commonly presented dialysis as the default treatment for advanced kidney disease and CKM as a subordinate option. Presenting conservative management and dialysis as on an equal footing enables patient to take a more active role in decision-making. These findings have important implications for clinical practice and education.

## Electronic supplementary material

Below is the link to the electronic supplementary material.

Supplementary Material 1

Supplementary Material 2

Supplementary Material 3

## Data Availability

Restrictions apply to the availability of study data, however requests to access the dataset can be made to the lead author (LES) 6 months after the study close date (30 November 2025).

## Abbreviations

CKM: Conservative kidney management
DOC: Doctor
NUR: Nurse
MC: My clinician
PAT: Patient
