# Supplementary material for: Communicating treatment options to older patients with advanced kidney disease: a conversation analysis study
Source: BMC Nephrol. 2024 Nov 21;25:417. doi: 10.1186/s12882-024-03855-w (PMC11580699; doi:10.1186/s12882-024-03855-w)
Supplement: Supplementary file 2 — Supplementary Material 2 [file 12882_2024_3855_MOESM2_ESM.docx]

**Supplemental Material**

Sample size

We aimed to record approximately 6 clinicians (depending on staff numbers and types) at each site (totalling c.24), using purposive sampling techniques to recruit a range of diverse clinicians, taking into account years of experience, age, gender and ethnicity. For each clinician, we aimed to record 4-6 consultations with eligible patients (and accompanying carers), resulting in 96-144 recorded consultations which were estimated to provide c.40-90 hours of data. This is comparable to that of previous successful conversation analytic studies in healthcare (e.g. VOICE: VideOing to Improve Communication Through Education^47^ used just under 50 hours). The number of recordings and participants was calculated to allow identification of recurrent practices used on multiple occasions by multiple individuals. The sample size also ensures we have a suitable number of clinical encounters to understand how treatment decision-making is discussed and supported in clinical practice across different renal units and clinician groups, and therefore that the findings from this research are not based on one participant’s idiosyncratic style of communicating, one clinician type or one patient type. All clinicians and patients who participate in a video-recorded consultation were invited to complete a questionnaire; we anticipated this would result in at least 80 responses, which is more than sufficient for exploratory analyses nested in a primarily qualitative study.

Screening and invitation

Clinicians were approached to participate by email by the renal unit clinical lead, with an attached PIS. Up to two follow up reminders were sent. If a clinician agreed to take part, a renal team member screened their upcoming clinic lists to identify eligible patients.

Patients who met the inclusion criteria were sent an introduction letter, participant information sheet and reply slip. Patients who opted in or requested more information were contacted by RS to answer any questions. Patients who did not reply were followed up by the screener. If no contact had been made, RS approached patients in the waiting room prior to their consultation to ask if they would like to find out more. Clinicians were notified where patients had opted in.

Screening – exclusions

Cases were excluded where: the patient had already decided on treatment (n=55); discussing the treatment options was not a primary activity in the conversation (when the decision was flagged as something to be discussed later (n=11), or the decision was not raised (n=9)); the conversation relied heavily on a previous conversation (n=11); or the clinician gave an explicit treatment recommendation (n=2), as these include different kinds of actions from option-listing and hence will be analysed separately.
