# Supplementary material for: Communicating treatment options to older patients with advanced kidney disease: a conversation analysis study
Source: BMC Nephrol. 2024 Nov 21;25:417. doi: 10.1186/s12882-024-03855-w (PMC11580699; doi:10.1186/s12882-024-03855-w)
Supplement: Supplementary file 3 — Supplementary Material 3 [file 12882_2024_3855_MOESM3_ESM.docx]

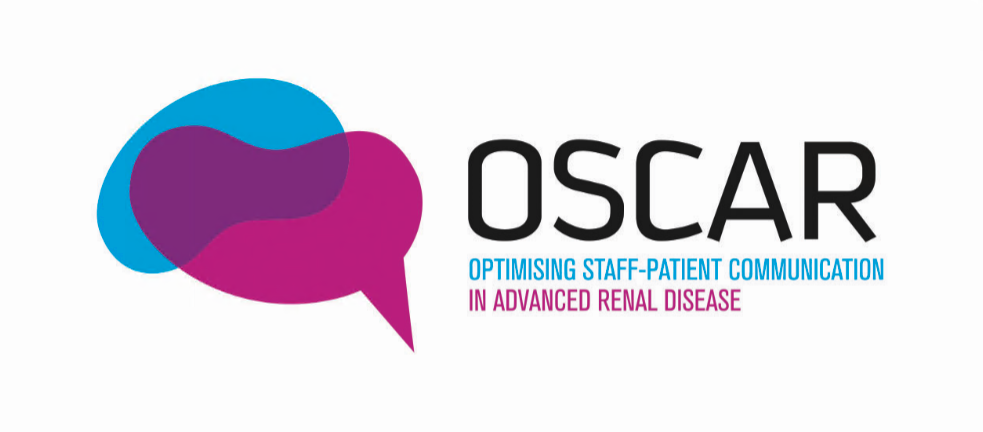

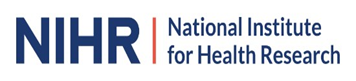

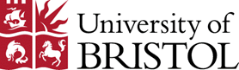
v

**SDM-Q-9 for patients**

When completing these questions, please think about your recent recorded consultation (whether with a doctor or nurse) and your decision which treatment to have for your kidney disease, if it gets worse.

Nine statements related to the decision-making in your consultation are listed below. For each statement please indicate how much you agree or disagree.

|  | **My clinician made clear that a decision needs to be made.** | | | | | |
| --- | --- | --- | --- | --- | --- | --- |
|  | Completely disagree | Strongly disagree | Somewhat disagree | Somewhat agree | Strongly agree | Completely agree |
|  | **My clinician wanted to know exactly how I want to be involved in making the decision.** | | | | | |
|  | Completely disagree | Strongly disagree | Somewhat disagree | Somewhat agree | Strongly agree | Completely agree |
|  | **My clinician told me that there are different options for treating my medical condition.** | | | | | |
|  | Completely disagree | Strongly disagree | Somewhat disagree | Somewhat agree | Strongly agree | Completely agree |
|  | **My clinician precisely explained the advantages and disadvantages of the treatment options.** | | | | | |
|  | Completely disagree | Strongly disagree | Somewhat disagree | Somewhat agree | Strongly agree | Completely agree |
|  | **My clinician helped me understand all the information.** | | | | | |
|  | Completely disagree | Strongly disagree | Somewhat disagree | Somewhat agree | Strongly agree | Completely agree |
|  | **My clinician asked me which tratement option I prefer.** | | | | | |
|  | Completely disagree | Strongly disagree | Somewhat disagree | Somewhat agree | Strongly agree | Completely agree |
|  | **My clinician and I thoroughly weighed the different treatment options.** | | | | | |
|  | Completely disagree | Strongly disagree | Somewhat disagree | Somewhat agree | Strongly agree | Completely agree |
|  | **My clinician and I selected a treatment option together** | | | | | |
|  | Completely disagree | Strongly disagree | Somewhat disagree | Somewhat agree | Strongly agree | Completely agree |
|  | **My clinician and I reached an agreement on how to proceed.** | | | | | |
|  | Completely disagree | Strongly disagree | Somewhat disagree | Somewhat agree | Strongly agree | Completely agree |

Adapted from: Scholl I, Kriston L, Dirmaier J, Buchholz A, Härter M. Development and psychometric properties of the Shared Decision Making Questionnaire - physician version (SDM-Q-Doc). *Patient Education and Counseling.* 2012;88(2):284-90.
